# Structure and Evolutionary Origin of Ca^2+^-Dependent Herring Type II Antifreeze Protein

**DOI:** 10.1371/journal.pone.0000548

**Published:** 2007-06-20

**Authors:** Yang Liu, Zhengjun Li, Qingsong Lin, Jan Kosinski, J. Seetharaman, Janusz M. Bujnicki, J. Sivaraman, Choy-Leong Hew

**Affiliations:** 1 Department of Biological Sciences, National University of Singapore, Singapore, Singapore; 2 International Institute of Molecular and Cell Biology, Warsaw, Poland; 3 X4 Beamline, Brookhaven National Laboratory, Upton, New York, United States of America; Vanderbilt University, United States of America

## Abstract

In order to survive under extremely cold environments, many organisms produce antifreeze proteins (AFPs). AFPs inhibit the growth of ice crystals and protect organisms from freezing damage. Fish AFPs can be classified into five distinct types based on their structures. Here we report the structure of herring AFP (hAFP), a Ca^2+^-dependent fish type II AFP. It exhibits a fold similar to the C-type (Ca^2+^-dependent) lectins with unique ice-binding features. The 1.7 Å crystal structure of hAFP with bound Ca^2+^ and site-directed mutagenesis reveal an ice-binding site consisting of Thr96, Thr98 and Ca^2+^-coordinating residues Asp94 and Glu99, which initiate hAFP adsorption onto the [10-10] prism plane of the ice lattice. The hAFP-ice interaction is further strengthened by the bound Ca^2+^ through the coordination with a water molecule of the ice lattice. This Ca^2+^-coordinated ice-binding mechanism is distinct from previously proposed mechanisms for other AFPs. However, phylogenetic analysis suggests that all type II AFPs evolved from the common ancestor and developed different ice-binding modes. We clarify the evolutionary relationship of type II AFPs to sugar-binding lectins.

## Introduction

Many species of ectothermic animals, plants, and microbes living in cold environments produce antifreeze proteins/polypeptides (AFPs) to protect them from freezing damage [1]–[4]. AFPs can lower the freezing temperature of a solution noncolligatively without affecting the melting temperature (thermal hysteresis), thus can prevent freezing of body fluids of AFP producing organisms. AFPs can also inhibit ice recrystallization, of which the large ice crystals grow with the expense of smaller ones, thus prevent cell damages during freeze-thaw cycles. It is generally accepted that AFPs function through adsorption of their flat ice-binding surfaces onto particular planes of ice crystals and prevent or inhibit further ice growth [2].

Antifreeze activity of AFPs attracts a lot of attention due to their wide potential commercial applications. They could be used in protection of economically important fishes and plants against frost or low temperatures. Several valuable aquaculture species like Atlantic salmon (*Salmo salar*) or goldfish (*Carassius auratus*) cannot survive in icy sea water, which poses severe limitations in culturing commercially important fishes in colder climates. Therefore a considerable effort is being undertaken to obtain transgenic fishes expressing functional AFPs. AFP genes have been successfully expressed in transgenic goldfish and salmon [5], [6]. However, the expression levels are low and additional studies are needed to obtain transgenic fishes with AFP expressed in proper tissues, sufficient expression level and activity. Rationally redesigned AFP gene from winter flounder was successfully expressed in spring wheat [7]. Spring wheat is highly susceptible to frost damage while transgenic wheat exhibited frost resistance even at temperatures as low as −7°C. Another application was found in food industry. Genetically modified type III AFP from ocean pout has already been introduced as a component of several ice cream brands produced by Unilever company. The protein is used to make ice creams smoother and creamier. These products are already on the market in USA, New Zealand and Australia [8]. Other proposed applications of AFPs are found in cryosurgery of tumours or transplantation and transfusion [9]. AFPs are also able to inhibit gas hydrate formation, thus showing great application potential in petroleum industry [10].

Fish AFPs are classified into antifreeze glycoproteins (AFGPs) and type I to IV AFPs based on their structural diversity [2]. Type II AFPs are 14–24 kDa cysteine-rich proteins, which have been found in five fish species, including herring (*Clupea harengus*), rainbow smelt (*Osmerus mordax*), Japanese smelt (*Hypomesus nipponensis*), sea raven (*Hemitripterus americanus*) and longsnout poacher (*Brachyopsis rostratus*)[2], [11]. They are the largest globular fish AFPs known to date, which are homologous to the C-type (Ca^2+^-dependent) lectin-like domains (CTLDs) [12], [13]. Homology modeling and NMR studies on type II AFPs indicated that these proteins share the same characteristic fold, which includes disulfide bridges, α-helices and β-sheets, as well as a large proportion of coil structure [12], [14], [15]. Both herring and two smelt AFPs depend on Ca^2+^ for their antifreeze activity, while the functions of sea raven and longsnout poacher AFPs are Ca^2+^ independent. Mutational analyses of sea raven AFP indicated that its ice-binding site, which is still undefined, is distinct from the Ca^2+^- and sugar-binding site of the lectins [16]. On the other hand, in herring AFP (hAFP), substitution of Ca^2+^ with other divalent metal ions decreased antifreeze activities and altered ice crystal morphologies [12], leading to the speculation that the Ca^2+^-binding site of hAFP is directly involved in ice binding. It is possible that Ca^2+^-dependent and -independent type II AFPs may have evolved distinct ice-binding mechanisms. To understand the Ca^2+^-dependent ice-binding mechanism, we have determined the X-ray structure of the Ca^2+^-dependent type II hAFP at 1.7Å resolution. Consistent with the structure, site-directed mutations disrupt the antifreeze activity. The structural basis for the ice-binding mechanism of hAFP is distinct from that of other known AFPs. A comprehensive sequence and phylogenetic analysis of hAFP with its homologs reveals that all type II AFPs evolved from a common ancestor and developed different ice-binding modes. The phylogenetic tree also suggests that ice-binding AFPs share their ancestor with fish skin mucus sugar-binding lectins, snake venom domain swapped CTLDs and the REG group of lectins.

## Results

### Overall structure

**Figure 1:**
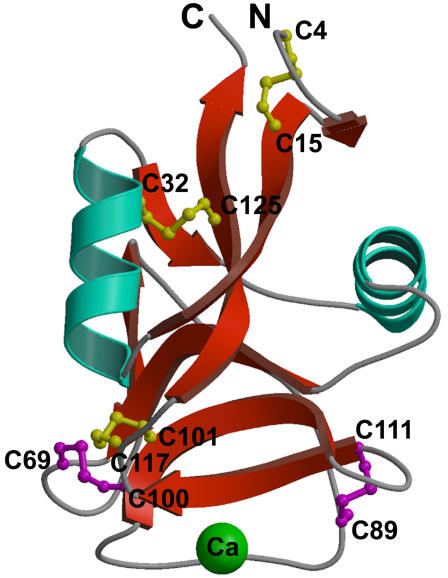
Ribbon representation of the hAFP structure. The positions of ten cysteine residues are labeled. Three conserved disulfide bonds (Cys4-Cys15, Cys32-Cys125, and Cys101-Cys117) are shown in yellow and two unique disulfides bonds (Cys69-Cys100 and Cys89-Cys111) in magenta. The Ca^2+^ ion is shown in green. This diagram was generated using the programs MOLSCRIPT and Raster3D [39], [40].

The crystal structure of hAFP, a first representative member of the Ca^2+^-dependent type II AFPs, has been determined (Figure 1; Table 1). hAFP crystallized with six molecules in the asymmetric unit. Each hAFP monomer consists of residues from Cys4 to Lys130. Neither the C-terminal His tag nor the N-terminal first three residues, had interpretable density and were not modeled. Gel filtration and dynamic light scattering experiments indicated that hAFP exists as a monomer in solution (data not shown). Each monomer comprises two twisted anti-parallel β-sheets with an α-helix on either side (Figure 1). The overall three-dimensional fold of hAFP resembles the long-form CTLDs and is different from other types of AFPs [13]. As expected, all 10 cysteines of hAFP are paired to form disulfide bonds, Cys4-Cys15, Cys32-Cys125, Cys69-Cys100, Cys89-Cys111 and Cys101-Cys117 (Figure 1). Three of the five disulfide bonds are conserved in the CTLDs. Remarkably, the additional two disulfide bonds (Cys69-Cys100 and Cys89-Cys111) are located at each end of the Ca^2+^-binding loop (Gln92-Asp114). Of the six prolines present in hAFP, only Pro93 is in the *cis* conformation and located in the ice-binding loop between two Ca^2+^-coordinating side chains and it may play a crucial role for the stability of the coordination sphere.

**Table 1:** Data collection, phasing and refinement statistics.

	Native	hAFP-Sm
**Data collection**		
Space group	P2_1_2_1_2_1_	P2_1_2_1_2_1_
Cell dimensions		
*a*, *b*, *c* (Å)	*31.28,146.42,192.41*	*31.27,146.70,192.85*
*α*, *β*, *γ* (°)	*90*, *90*, *90*	*90*, *90*, *90*
Resolution range (Å)	50.0–1.7 (1.76–1.7)	50.0–2.2 (2.28–2.2)
*R*_sym_	5.8 (39.4)	9.6 (18.4)
*I*/σ*I*	12 (7.4)	15.1 (6.8)
Completeness (%)	96.5 (91.2)	99.9 (99.7)
Redundancy	12.9 (7.1)	12.3 (5.5)
**Refinement**		
Resolution range*(Å)	20–1.7	20–2.2
No. reflections	90464	81869
*R*_work/_ *R*_free_	0.209/0.24	0.229/0.259
No. atoms		
Protein	5976	5976
Ligand/ion	6	6
Water	581	471
B-factors		
Protein	19.3	18.5
Ligand/ion	25.0	16.7
Water	28.6	21.8
R.m.s deviations		
Bond lengths (Å)	0.006	0.007
Bond angles (°)	1.5	1.6

Values in parentheses are for the highest resolution shell.

Studies of the CTLDs revealed a broad diversity of functions for this molecular fold. Apart from Ca^2+^-dependent carbohydrate binding, the CTLDs have been reported to bind noncarbohydrate ligands, such as proteins, lipids, CaCO_3_ and ice [13]. The superpositions of all the structural homologs of hAFP were manually done using the program O [17] and show that they all adopt a similar CTLD fold except in the ice-binding loop region. Carbohydrate affinity chromatography experiments showed that hAFP could not bind to carbohydrates (data not shown). The closest structural similarity of hAFP is with lithostathine (pdb code: 1qdd; RMSD = 1.7 Å for 122 Cα atoms). This is followed by the Ca^2+^-dependent sugar-binding lectins such as a mannose-binding protein (pdb code: 1sl6; RMSD = 2.2 Å for 124 Cα atoms). Lithostathine, which is one of the CTLD-containing proteins, inhibits the growth of calcite crystals, a function similar to the hAFP ice crystal growth inhibition; using a completely different region, *i.e.* through the N-terminal domain [18]. However, no ice-binding activity could be detected in lithostathine [19].

### Sequence comparisons and phylogenetic analysis

**Figure 2:**
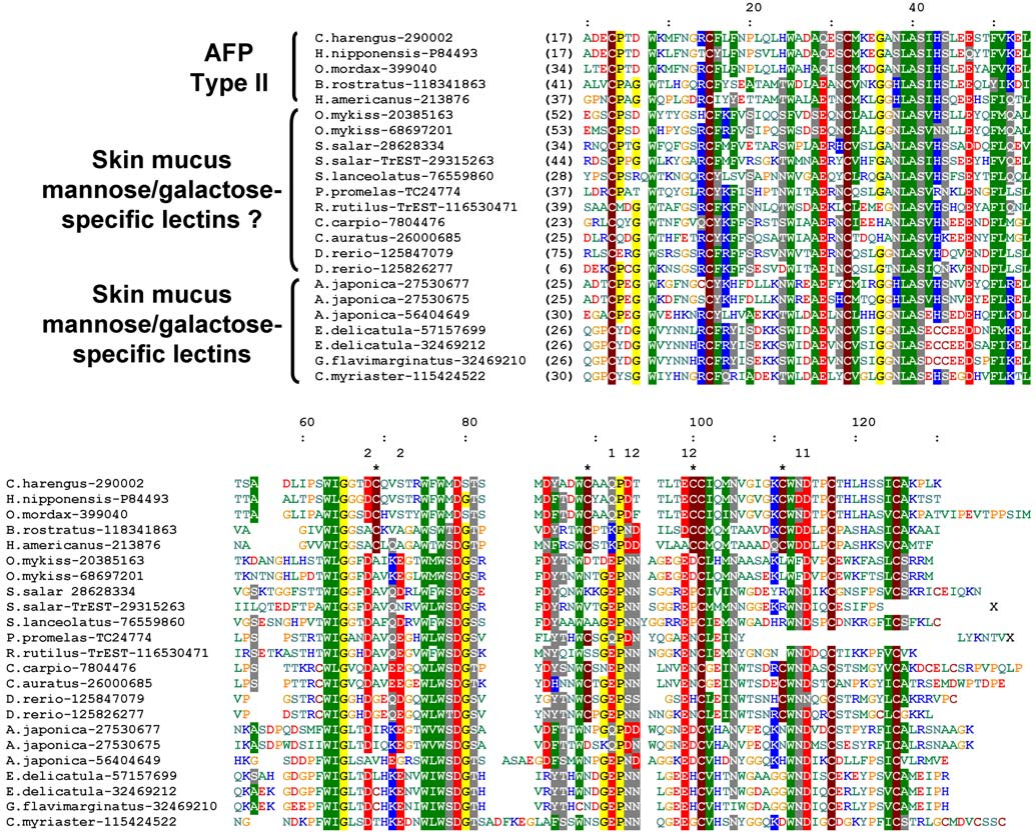
Sequence alignment of type II AFPs and most closely related fish-specific lectins. Similar or identical amino acid residues are highlighted. Residues are colored according to their physicochemical properties. Numbering in the top ruler corresponds to residue numbering of hAFP. Numbers in brackets in the N- and C-terminal part of the alignment indicate how many amino acids have been omitted for the sake of the clarity. Type II AFPs–specific cysteines forming disulfide bonds are indicated by “*”. Residues forming the first Ca^2+^-binding site in hAFP are indicated with “1”. Residues homologous to residues of the second Ca^2+^-binding site are indicated as “2”.

**Figure 3:**
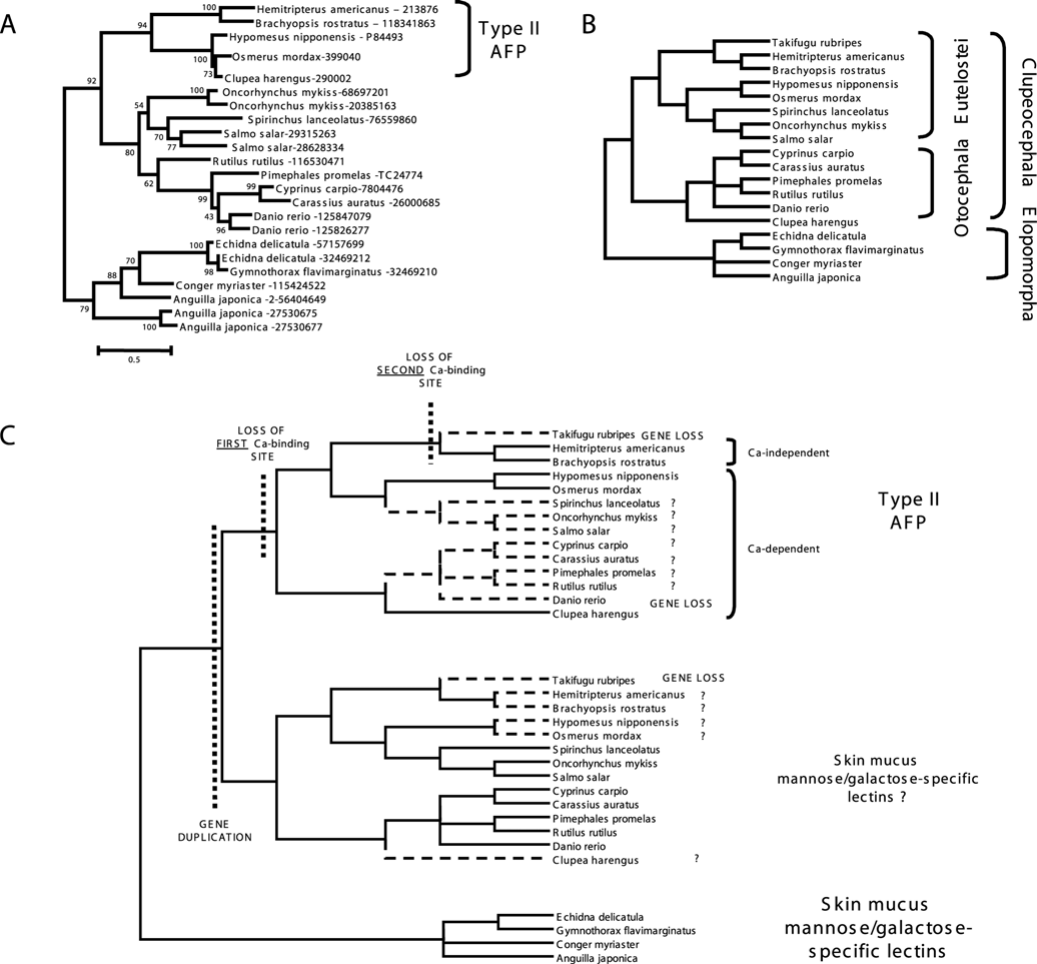
Phylogenetic and evolutionary analyses of type II AFPs. (A) Maximum likelihood (ML) phylogenetic tree of type II AFPs and representatives of the most closely related fish skin mucus lectins. The values on the nodes represent the bootstrap support for the individual branches. (B) Phylogenetic tree of Teleostei reproduced based on Lundberg, John G. 2006. Teleostei. Version 18 August 2006 (temporary). http://tolweb.org/Teleostei/15054/2006.08.18 in The Tree of Life Web Project, http://tolweb.org/ and classification from the NCBI Taxonomy database (http://www.ncbi.nlm.nih.gov/Taxonomy/). (C) Evolutionary history of type II AFPs. Putative gene losses are indicated. Those taxa for which gene losses could not be stated due to insufficient genomic data are indicated with “?”.

Multiple sequence alignment of hAFP and homologous proteins, including other type II AFPs and fish sugar-binding lectins from skin mucus, reveals that type II AFPs are distinguished from other CTLDs by two additional disulfide bonds (Figure 2). One of these disulfides is present in lectin sequences from zebrafish (*Danio rerio*), carp (*Cyprinus carpio*) and goldfish (*Carassius auratus*), which belong to the Cyprinidae family of fishes. A phylogenetic tree calculated from the sequence alignment (Figure 3A) reveals that all type II AFPs (Ca^2+^-dependent and independent) form a single branch among lectins, indicating that they evolved from a common ancestor. Moreover, they clearly group together with fish-specific lectins that are known to bind mannose or galactose, and locate in skin mucus. They have been implicated in antibacterial defense, most probably owing to their ability to bind carbohydrates in bacterial cell walls. Most of these lectins bear the EPN sequence motif that is typically associated with a mannose-binding specificity, while those involved in galactose binding exhibit a QPD motif at the same position. Interestingly, Ca^2+^-dependent type II AFPs also exhibit the QPD motif. Both EPN and QPD motifs contain residues responsible for binding of Ca^2+^ ion. Analysis of a multiple sequence alignment reveals that several other residues responsible for Ca^2+ ^binding are conserved among fish skin mucus lectins and only a few are conserved among type II AFPs. Most sequences of fish skin mucus lectins contain all residues required for binding of two Ca^2+^ ions near the carbohydrate-binding site (Figure 2), similarly to other lectins. Ca^2+^-dependent type II AFPs contain all residues required to bind one Ca^2+^ ion (Q92, D94, E99, E113 and D114). Only one residue (D68) of the second Ca^2+^-binding site is conserved between Ca^2+^-dependent type AFPs and other lectins. In Ca^2+^-independent type II AFPs, most of the Ca^2+^-binding residues are lost, which explains their Ca^2+^-independent ice-binding activity.

A reconciliation of a phylogenetic tree (Figure 3C) of type II AFPs and fish specific lectins from skin mucus (Figure 3A) with an evolutionary tree of teleost fishes from the Elopocephala group (*i.e.* a group of Teleostei in which type II AFPs are found) (Figure 3B) reveals that type II AFPs originated from an ancestral duplication of a gene encoding a mannose or galactose-binding lectin. The duplication probably occurred in the ancestor of Clupeocephala, since no type II AFP gene was found outside that group. The most parsimonious explanation of the data is that one of the duplicated genes developed a new function of ice binding before differentiation of Otocephala and Euteleostei groups from the ancestral Clupeocephalan (Figure 3C). Notably, we could not find a gene coding for type II AFPs in two organisms with almost completely sequenced genomes: *Danio rerio* and *Takifugu rubripes* (the latter lacks also a gene belonging to the paralogous family of fish-specific lectins described here). Apparently, the genes were lost from the genomes of these fishes, which are known for having experienced multiple genome duplications and multiple gene losses [20]–[22]. However, genomes have not been completely sequenced for most of the organisms analyzed here and therefore we must avoid speculating in which organisms the genes of type II AFPs were lost.

**Figure 4:**
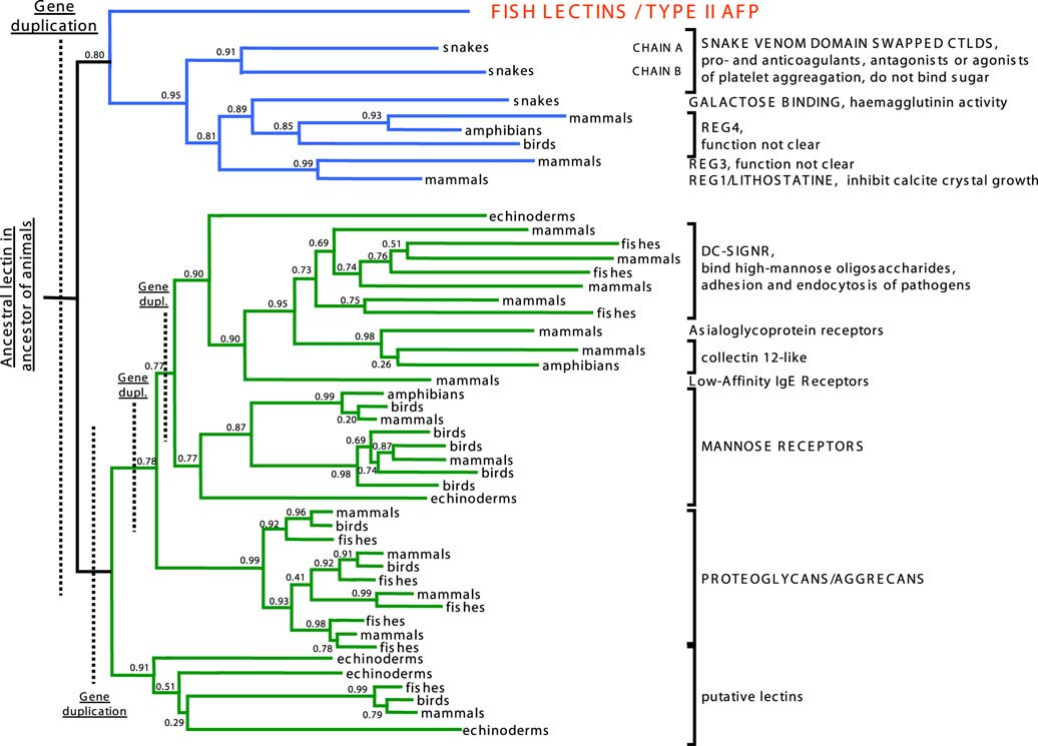
Maximum likelihood (ML) phylogenetic tree of type II AFPs, fish skin mucus lectins and other closely related lectins. Values at the nodes represent branch support (probability values) derived with parametric approximate likelihood ratio test for branches (aLRT). A clade postulated in this work to be monophyletic and containing type AFPs is shown in blue. Other clades are shown in green. The position of the root of the tree is hypothetical. Gene duplications responsible for a rise of major clades are indicated.

To reveal the evolutionary origin of fish skin mucus lectins and type II AFPs family, we constructed a phylogenetic tree containing other closely related lectin families (Figure 4) including lectins REG group (which includes lithostathine), snake-specific lectins, DC-SIGN receptors, mannose receptors and others. The most closely related families are snake venom domain swapped CTLDs, snake galactose-binding lectins and REG group. Phylogenetic analysis (Figure 4) suggests that skin mucus lectins and type II AFPs share a common ancestor with snake specific lectins and lectins from REG group. This common ancestor could have arisen by a duplication of a gene encoding a galactose- or mannose-binding lectin. In different animals the new gene underwent subfunctionalization and in different phyla it acquired class specific functions. In teleost fishes it underwent additional duplication and gave rise to type II AFPs and skin mucus lectins. Similar duplications and subfunctionalizations occurred in snakes and animals (giving snake venom lectin families and different families of REG group).

Type II AFPs evolved several features distinct from other lectins. They contain two additional disulfide bonds and Ca^2+^-independent type II AFPs lost Ca^2+^-binding sites. The Ca^2+^-binding sites must have degenerated in the ancestor of Neognathi (*i.e.* the group with Ca^2+^-independent type II AFPs from *Hemitripterus americanus* and *Brachyopsis rostratus*) (Figure 3C). It is not clear when the two additional disulfide bridges appeared. They might have been present already in the last common ancestor (LCA) of type II AFPs and fish specific lectins, because some of fish skin mucus lectins (from Cyprinidae family of fishes) contain cysteines from one additional disulfide bridge (Figure 2). According to this hypothesis skin mucus lectins from Cyprinidae would have lost one disulfide bridge and other fish skin mucus lectins would have lost both. However, it is also possible that additional disulfide bridges appeared in the last common ancestor of type II AFPs and a disulfide bridge evolved in Cyprinidae independently.

Our evolutionary analysis explains why type II AFPs genes were found in distantly related organisms (Atlantic herring belongs to Otocephala while all other type II AFP genes were found only in organisms from Euteleostei group). We propose that all type II AFPs arose by duplication and subfunctionalization of the same ancestor teleost fish lectin, contrary to the previous suggestion that they arose by convergent evolution [2].

### Ca^2+^-binding site

**Figure 5:**
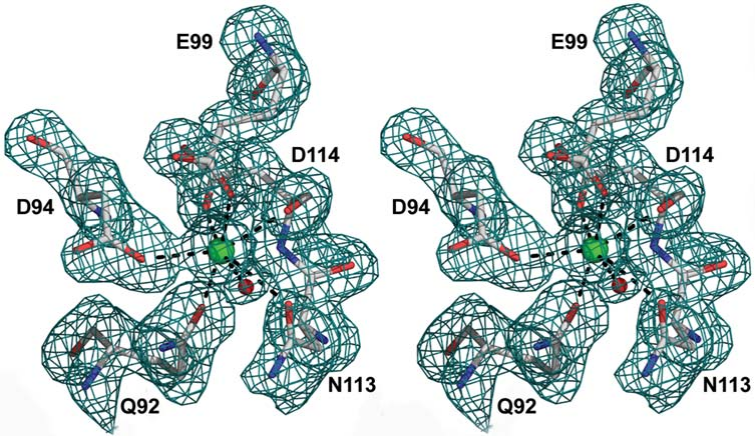
Stereo view of Ca^2+^-coordination sphere. Black dashed lines indicate the coordination bonds. The Ca^2+^ ion (green) is coordinated with a water molecule (red), Gln92 O^ε1^, Asp94 O^δ2^, Glu99 O^ε1^, Asn113 O^δ1^ and Asp114 O and O^δ1^. This diagram was generated using PyMOL [41].

Sequence analysis suggested that hAFP contains one conserved Ca^2+^-binding site that is present in the CTLDs [13]. In the electron density map of the native protein, there is a strong peak corresponding to a metal ion. Based on the coordination geometry and the type of liganding side chains, it was interpreted as the Ca^2+^ ion. It is worth mentioning that the crystallization conditions contained 1 mM of Ca^2+^. The bound Ca^2+^ is coordinated by Gln92 O^ε1^, Asp94 O^δ2^, Glu99 O^ε1^, Asn113 O^δ1^ and Asp114 O and O^δ1^ (Figure 5). The pentagonal bipyramidal coordinating sphere for Ca^2+^ is completed by a water molecule. Presumably when hAFP interacts with the ice crystal, this water molecule may be incorporated into the growing ice lattice. Previous mutagenesis study of Ca^2+^-coordinating residues suggested that the loop region defined by Glu99 and Asp114 might be the ice-binding site [23]. However, the crystal structure indicates that the side-chain of Asp114 is not exposed on the protein surface and therefore it is unable to directly interact with ice. But the main-chain and side chain oxygen atoms of Asp114 forms a part of the Ca^2+^coordination sphere, any substitution of Asp114 will disrupt the Ca^2+^ coordination. The important role of Asp114 is to stabilize the Ca^2+^-binding loop and Ca^2+^ coordination rather than being directly involved in ice binding (Figure 5).

### Ice-binding site and antifreeze activity

**Figure 6:**
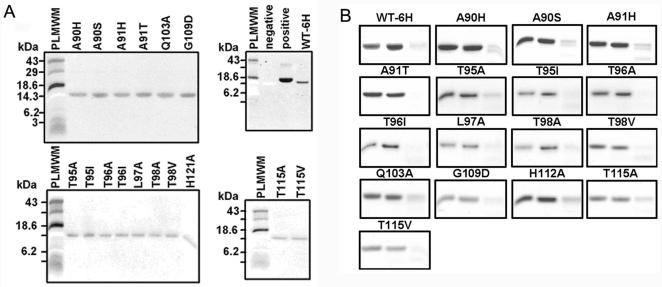
Ca^2+^-binding properties of hAFP and its mutants. (A) ^45^CaCl_2_ overlay assay of WT-6H and its mutants. Lysozyme was used as negative control and β-lactoglobulin was used as positive control. PLMWM represents prestained low molecular weight marker. Both wild-type hAFP and its mutants can bind Ca^2+^ ions properly. (B) Proteolysis protection assay of WT-6H and its mutants. Endoprotease Glu-C was used to detect conformational changes of hAFP and its mutants as modulated by Ca^2+^ ions. In the absence of Ca^2+^ ions, hAFPs were subjected to Glu-C cleavage. Three lanes of each sample from left to right represent hAFP, and hAFP treated with Glu-C, respectively, in the presence, and in the absence, of Ca^2+^ ions.

**Figure 7:**
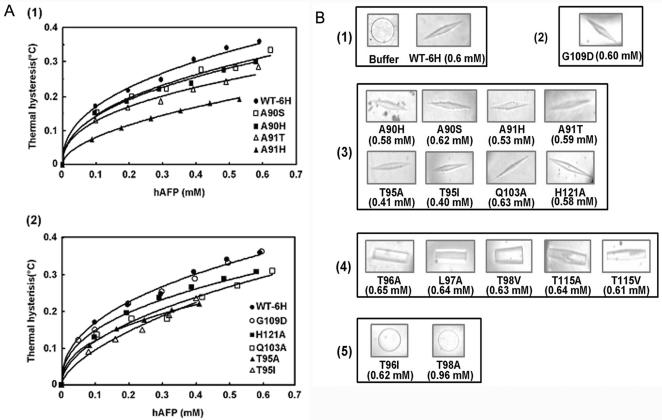
Thermal hysteresis activities and ice crystal morphologies of wild-type hAFP (WT-6H) and its mutants. (A) Concentration dependent thermal hysteresis activity of (1) WT-6H (•), mutants Ala90Ser (□), Ala90His (▪), Ala91His (▴), and Ala91Thr (▵), and (2) WT-6H (•), mutants Thr95Ala (▴), Thr95Ile (▵), Gln103Ala (□), Gly109Asp (○), and His121Ala (▪), was measured as described under “Materials and Methods” using a Clifton nanoliter osmometer. Values showed represent means of triplicate measurements done on each single sample. (B) Ice crystal morphologies of hAFP and its mutants. Samples were: (1) buffer alone and wild-type hAFP; (2) the mutant that exhibited no effect on thermal hysteresis; (3) mutants that showed reduced thermal hysteresis activities; (4) mutants which retained the ability to modify the ice crystal with no detectable thermal hysteresis activities, (5) mutants that exhibited complete loss of antifreeze activity. The protein concentration used for each sample is also indicated.

To locate the ice-binding surface of hAFP, structure-based site-directed mutagenesis was carried out at the non-Ca^2+^-coordinating residues in the Ca^2+^-binding loop region. Ala90, Ala91, Thr95, Thr96, Leu97, Thr98, Gln103, Gly109, Thr115, and His121 were selected for the mutation (Table S1). These mutants were characterized with respect to their antifreeze activity and Ca^2+^-binding properties. The ^45^CaCl_2_ overlay assay and proteolysis protection analysis (Figure 6) confirmed the Ca^2+^-binding property of the mutants. Further, the Ca^2+^ affinity analysis on selected mutants showed that they had similar K_d_ to the wild-type hAFP (Table S2). The spectra of Ca^2+^-dependent intrinsic fluorescence (Figure S1) indicated that there are no significant changes in their properties between the wild-type hAFP and the mutants. However, thermal hysteresis activities of several mutants were dramatically reduced (Figure 7, Table S3 and Table 2). Our results showed that mutating Gly109 to Asp had no effect on antifreeze activity. This suggests that this residue is not close to the ice-binding site. Mutants of Ala90, Ala91, Thr95, Gln103, and His121 largely retained antifreeze activity, suggesting that they were less critical for ice binding. However, substitution of Ala 91 with His only retained 56% of the activity of wild-type hAFP and altered ice crystal morphology, indicating certain steric hindrance by the bulky side chain. This is consistent with our ice-binding model (as discussed below) showing that Ala91 is close to the ice-binding site and the ice surface. Whereas in the case of Thr at 96, 98 and 115, which are in the proximity of Ca^2+^-coordinating residues E99 and D114, mutating these residues to Ala caused significant loss of thermal hysteresis activity, implying that the removal of any possible hydrogen bonds may disrupt the hAFP-ice interaction. Similarly, the replacement of Leu at position 97 with Ala also led to a catastrophic reduction of thermal hysteresis.

**Table 2:** Relative thermal hysteresis activities of hAFP mutants (0.4 mM) compared with that of the wild type hAFP WT-6H.

Protein name	Thermal hysteresis activity
WT-6H	100%
A90H	85%
A90S	87%
A91H	56%
A91T	75%
T95A	72%
T95I	74%
Q103A	79%
H121A	87%

Ice crystal morphology is also indicative of antifreeze activity. In the absence of AFPs, the ice crystal grows as a circular disk (Figure 7B(1) buffer). In the presence of AFPs, there are different forms of ice crystals due to the binding of AFPs to different prism faces of ice and their different binding capabilities. Ice crystal morphologies of these mutants varied from hexagonal bipyramidal forms with the same or reduced activities compared to the wild-type hAFP, to columnar spicules with weak ice-binding activity without detectable thermal hysteresis. In extreme cases, ice crystals of mutants Thr96Ile and Thr98Ala are circular plates similar to the mock control, indicating the complete loss of the ice-binding affinity (Figure 7B(5)).

**Figure 8:**
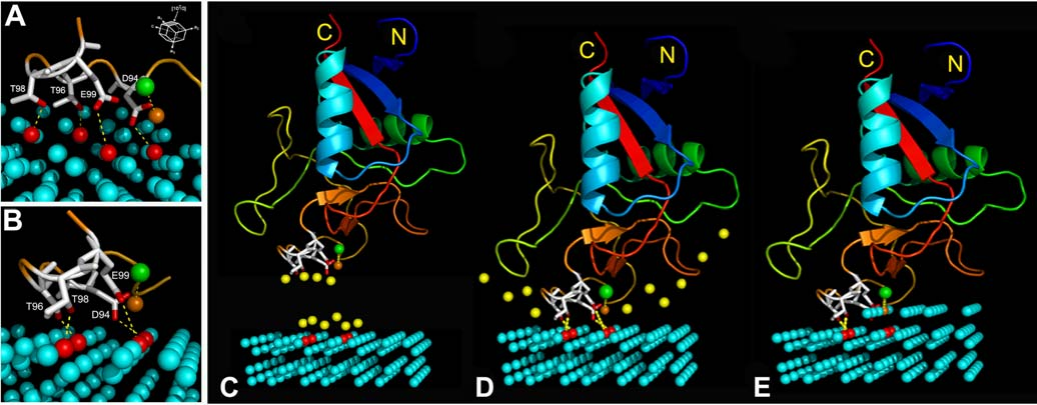
A model for hAFP binding to [10-10] prism plane of ice. (A) and (B) show that four ice-binding residues Asp94, Thr96, Thr98, and Glu99 form hydrogen bonds with water molecules of the ice lattice (highlighted in red) through respective side-chain oxygen atoms as indicated by yellow dotted lines. The Ca^2+^ ion is shown as a green sphere. The water molecule coordinating with the Ca^2+^ ion is shown as an orange sphere. The orientation of the ice lattice and the [10-10] prism plane are indicated. (C) Yellow spheres represent the water molecules constrained at the hAFP ice-binding face and the ice-water interface. (D) When hAFP binds to ice, constrained water molecules are released. This is considered as an entropy favorable process. (E) With the growth of the second layer of ice on the prism plane, the Ca^2+^-coordinating water molecule is incorporated into the ice lattice, thus the hAFP-ice interaction is further stabilized. This diagram was generated using PyMOL [41].

Altogether, mutational results indicate that residues Thr96, Leu97, Thr98, and Thr115 are most critical for antifreeze activity. All these residues are in the vicinity of the bound Ca^2+^ ion. Considering both the structural data and results of site-directed mutagenesis obtained here, together with the previous mutagenesis study of Ca^2+^-coordinating residues Asp94 and Glu99, which severely hampered the ice binding [23], we propose that the ice-binding site of hAFP consists of Asp94, Thr96, Thr98, and Glu99 that form a relatively flat surface to interact with ice (Figure 8AB). Further, the hAFP-ice interaction is strengthened by the coordinating Ca^2+^ ion. Our results also provide additional evidence to claim that the ice-binding mechanisms of hAFP and sea raven AFP are quite different. In hAFP, Thr98Ala mutant was inactive, whereas in sea raven AFP the corresponding residue is an Ala residue.

The crystal structure of hAFP shows that Leu97 is not projected to the ice-binding surface. However, replacement of Leu with Ala has led to a significant reduction of thermal hysteresis, suggesting that this hydrophobic residue is vital to position its adjacent ice-binding residues Thr96 and Thr98 for interaction with the ice surface. Similarly, Thr115 is structurally important by forming a hydrogen bond with Pro116. Mutating this residue to Ala may affect the Ca^2+^ coordination with Asp114 and, thereby, lead to a decreased antifreeze activity. Mutating Gly109 to Asp had no effect on the antifreeze activity, in agreement with the structure showing that this residue is far away from the ice-binding site.

### Ice-binding model

The manual docking of the ice-binding surface of hAFP to various ice lattice planes of different arrays of water molecules (Figure S2) using the program O [17] suggests that hAFP prefers to bind to the [10-10] prism plane (Figure 8). Only with this plane all four ice-binding residues Asp94, Thr96, Thr98 and Glu99 can form hydrogen bonding contact with the ice-lattice. This model shows no steric clashes between hAFP and the ice-lattice. The inhibition of ice crystal growth by hAFP is primarily through the interactions of these residues and the direct Ca^2+^ coordination with the ice crystal lattice. Residues that bind to the ice lattice lie on a nearly flat surface, which may also accept small backbone conformational changes to interact more effectively with the ice lattice surface and inhibit ice crystal growth. The two additional disulfide bonds on both sides of the Ca^2+^-binding loop stabilize this surface and project the ice-binding residues to engage with the ice lattice. Since the ice-binding site is stabilized in solution, there should not be much entropy loss when it binds to the ice surface. On the other hand, some water molecules at the flat ice-binding protein surface, as well as at the ice-water interface, are partially ordered (Figure 8C). When hAFP binds to ice, these ordered water molecules are released. Thus, the binding of hAFP to the ice prism plane is probably an entropy favorable process (Figure 8D). This interaction is further strengthened when the Ca^2+^-coordinating water molecule is integrated into the ice lattice during the growth of second layer of water molecules on the ice crystal surface (Figure 8E).

## Discussion

We have determined the crystal structure of hAFP and identified its ice-binding site through structure based mutagenesis. This is the first crystal structure report for the type II AFPs. The Ca^2+^-dependent ice-binding mechanism we proposed may represent a common mechanism for other Ca^2+^-dependent AFPs, including other fish type II AFPs and a bacterial AFP identified to date [24].

The ice-binding mechanism described here is distinct from the mechanism proposed for other structurally characterized AFPs such as fish type I and III, and insect AFPs, which have much larger ice-binding surfaces compared to that of hAFP. Thus, surface complementarity and van der Waals interaction as proposed in the mechanism of other AFPs may not be main determinants for hAFP-ice interaction [25].

For type II AFPs, two disulfide bonds are critical to stabilize the ice-binding surface in the solution, while the sugar-binding activity may require the binding site to be more flexible, hence disfavoring the presence of multiple disulfide bonds. The reason that hAFP did not evolve a large flat ice-binding site with perfect ice lattice match may be due to the involvement of the Ca^2+^ cofactor, which provides sufficient binding force through coordination of water in the ice lattice. We propose that this ice-binding mechanism evolved from a sugar-binding mechanism of lectins, which relies on the coordination of sugar hydroxyl groups by the Ca^2+^ cofactor. Ca^2+^-independent type II AFPs have most likely evolved by developing a potentially larger flat ice-binding surface similar to that of other types of AFPs, that ensured strong interactions with the ice lattice even in the absence of the Ca^2+^ ion. Our data do not support the earlier hypothesis that Ca^2+^-independent and Ca^2+^-dependent type II AFPs evolved by convergent evolution. Instead, Ca^2+^-independent type II AFPs appear to have lost their Ca^2+^-binding sites, while preserving the ice-binding activity.

Summarizing, our combined structural, biochemical and bioinformatics analyses provide a roadmap for sequence-structure-function relationships among AFPs and suggest a scenario for their evolution. Considering the fact that type II AFPs are commercially important proteins, our results may aid in rational engineering of AFPs with improved features.

## Materials and Methods

### Cloning, expression and purification

The hAFP gene excluding the signal sequence was PCR amplified from hAFP cDNA. To facilitate the purification, a (His)_6_-tag was introduced at the C-terminus. The PCR products were inserted into the pGAPZα A vector (Invitrogen, San Diego, CA) for yeast expression [26]. The resulting construct was verified by DNA sequencing. Cells were grown at 23C for 2 days before harvest. After centrifugation, the secreted hAFP was precipitated by incubation with 31.3 g ammonium sulfate per 100 ml of culture medium at 4C overnight. The ammonium sulfate precipitate was resuspended in 20 mM Tris-HCl, pH 8.0 and purified by Ni-NTA chromatography following the manufacturer's recommendation (Qiagen, Valencia, CA). The bound protein was eluted with 300 mM imidazole in 20 mM Tris-HCl, containing 0.5 M NaCl, pH 8.0 and desalted with a Sephadex G-25 column. Further purification was performed with hydrophobic interaction chromatography using Phenyl Sepharose column (GE Healthcare, Singapore). A linear gradient of 1.8-0 M ammonium sulfate in 20 mM Tris-HCl, pH 8.0 was used to elute the protein. As a final step the protein was passed through the Superdex 75 gel filtration column (GE Healthcare) with a buffer consisting of 20 mM Tris-HCl, pH 8.0, 100 mM NaCl and 1 mM CaCl_2_.

### Crystallization and data collection

hAFP was crystallized by the hanging-drop vapor-diffusion method at 298 K. Initial crystallization screening was performed using crystal screens 1 and 2 from Hampton Research. The crystallization drop contains 1 µl of protein solution (10 mg/ml) and 1 µl of reservoir solution. The drops were equilibrated against 1 ml of the reservoir solution. The best diffraction quality crystals were obtained with the reservoir solution containing 180 mM ammonium sulfate, 100 mM sodium acetate pH 4.5, 1 mM calcium chloride, 20% PEG 4000 and 3% D-glucose. For heavy atom derivatives, initial screen was performed using Hampton Research heavy atom screen kit. The best phases obtained from 1 mM samarium chloride (SmCl_3_) soaked for 12 hours. Prior to the data collection, the SmCl_3_ soaked single crystal was transferred to the cryo-protectant solution (mother liquid supplemented with 20% glycerol and 1 mM SmCl_3_), and then frozen at 100 K in a nitrogen gas cold stream (Oxford Cryosystems, Oxford, UK). Synchrotron data sets were collected at X12C beamline, NSLS, Brookhaven National Laboratory, USA. Diffraction data were processed using the program HKL2000 [27].

### Structure determination and refinement

The initial phases were calculated from the 2.2 Å Sm-SAD dataset which showed the strong anomalous signal. The asymmetric unit consists of six hAFP molecules. The single Ca^2+^-binding site of each hAFP was occupied by a Sm^3+^ ion. A total of six Sm^3+^ ions were identified using the program SOLVE [28]. Subsequent density modification and model building using the program SHARP and wARP enabled the tracing of the main chain atoms for up to ∼55% of the model [29]. The remaining parts of the model were manually built using the program O and refined with the program CNS [17], [30]. The final resolution was extended to 1.7 Å using a high resolution native dataset and refined without NCS. Statistics for data processing and refinement are listed in Table 1. Validation of the model was performed using the program PROCHECK [31]. The Ramachandran plot indicates that all the residues are in the most favored and allowed regions except for Ser56 from each monomer (only for two monomers), which is located in the tight turn, but well defined by the electron density map.

### Construction and characterization of hAFP mutants

All mutants were generated using the PCR method following the procedure described previously (primer sequences are provided in Table S1) [23]. The PCR products containing the full-length sequences of hAFP mutant genes were subcloned into the pGAPZα A vector (Invitrogen). Positive mutant clones were confirmed by dideoxy sequencing. Protein expression and purification of wild-type hAFP (WT-6H) and its mutants followed the procedures described previously [26].

### Thermal hysteresis measurement and ice crystal morphology

AFP activity is measured by thermal hysteresis, which is defined as an observed difference in the melting and freezing points of ice in an aqueous solution. Thermal hysteresis is calculated by subtracting the freezing point from the melting point of a solution. The activity of wild-type hAFP and its mutants was measured by using a nanoliter osmometer (Clifton Technical Physics, Hartford, NY) as previously described [32]. Each sample was performed in triplicate from three different sample wells. All measurements were made in 40 mM Tris-HCl and 50 mM CaCl_2,_ pH 7.5. Ice crystal morphology was captured by video microscopy.

### Ca^2+^-binding ability detected by ^45^CaCl_2_ overlay

hAFPs (1–2 µg) were run on the nonreducing tricine SDS-PAGE and electrophoretically transferred to 0.2 µm nitrocellulose membranes. The blots were washed and then labeled with ^45^CaCl_2_ according to the procedure of Maruyama *et al*. [33]. The membranes were air dried and autoradiographed. After autoradiography the membranes were stained with ponceau S (data not shown).

### Proteolysis protection assay

Ca^2+^-inducted proteolysis protection assay was performed on recombinant hAFPs in 20 mM Tris-HCl, pH 8.0, with 1 mM CaCl_2_ or 5 mM EDTA, containing 0.2 mg/mL endoproteinase Glu-C for 1 h at 21°C [12]. After digestion, the reaction mixtures were resolved on the nonreducing tricine SDS-PAGE and stained with Coomassie Brilliant Blue.

### Ca^2+^-binding affinity analysis

After reverse-phase C4 HPLC purification, hAFP became inactive due to the removal of the Ca^2+^ ion. All buffers used for experiments with the apo-hAFP were treated with a Chelex 100 column, pH 7.5 (Bio-Rad Laboratories, Mississauga, Ontario, Canada) to become metal-free and stored in plastic tubes. Equilibrium dialysis of hAFP was performed with dispo-equilibrium dialyzers as described [23]. The protein concentrations of apo-hAFP and its mutants were shown in Table S2. Nonspecific binding was estimated and subtracted by using lysozyme as a substitution of hAFP.

### Intrinsic fluorescence of hAFP mutants

Steady state fluorescence of hAFP mutants was measured at room temperature using a QM-1 fluorescence spectrophotometer (Photon Technology International, Lawrenceville, NJ) as previously described [26]. Spectra of buffer only or buffer with Ca^2+^ ions were used to correct for light scattering. For the Ca^2+^-modulated conformational studies, spectra of apo-hAFPs in 40 mM Tris-HCl, pH 7.5, with or without 50 mM CaCl_2_, were achieved. Relative fluorescence intensity was obtained by using the maximum fluorescence intensity of hAFP in the absence of the Ca^2+^ ion as 100%. The protein concentrations used were within the range from 0.56–0.64 µM.

### Sequence analysis

The sequence of herring AFP was used as a query in PSI-BLAST searches of the non-redundant (nr) database of protein sequences at the NCBI with E-value threshold of 1e^-3^ [34]. After 10 iterations all sequences with E-value <10 were collected (yielding over 4,700 proteins). This number was reduced to 1,500 sequences with identity <70% using CD-HIT [35]. All these sequences were clustered using CLANS to identify true homologs of AFPs (220 sequences with higher similarity to each other than to any other protein family in the dataset) [36]. A multiple sequence alignment (MSA) of these sequences was built using MUSCLE and refined manually [37]. The MSA was used to infer a maximum likelihood (ML) phylogenetic tree using PHYML [38], from which the sequences forming a branch with type II AFPs were extracted. At this stage the closely related homologs of these sequences, which were previously removed at the 70% identity threshold, were returned to the analysis, giving a group of 172 type II AFP homologs. A new ML tree was constructed using the WAG model of amino acid substitution, followed by the parametric approximate likelihood ratio test (aLRT) to assess the significance of individual branches. For the clade containing type II AFPs and fish skin mucus lectins, a separate ML tree was constructed using the WAG model, and a bootstrap test with 100 replications has been applied to test its robustness.

## Supporting Information

Table S1 Antisense mutagenic primers of the mutants of hAFP
(0.04 MB DOC)

Table S2 Equilibrium dialysis assay of hAFP and its mutants
(0.03 MB DOC)

Table S3 Thermal hysteresis of solutions in the presence of hAFPs
(0.07 MB DOC)

Figure S1 Ca^2+^-induced intrinsic fluorescence changes of hAFP and its Ala
(0.18 MB DOC)

Figure S2 Lattice planes of ice Ih, the hexagonal crystal form of ordinary ice.
(0.07 MB DOC)
