# Supplementary material for: Structure and Evolutionary Origin of Ca2+-Dependent Herring Type II Antifreeze Protein
Source: PLoS One. 2007 Jun 20;2(6):e548. doi: 10.1371/journal.pone.0000548 (PMC1891086; doi:10.1371/journal.pone.0000548)
Supplement: Table S1 — Antisense mutagenic primers of the mutants of hAFP (0.04 MB DOC) [file pone.0000548.s001.doc]

**SUPPORTING INFORMATION**

**Table S1.** Antisense mutagenic primers of the mutants of hAFP

| **Protein name** | **Primer sequence** | **Restriction enzyme** |
| --- | --- | --- |
| A90H | AGTATCAGGTTGTGCATGGCACCAGTCAGCATA | - |
| **A90S** | AGTATCAGGTTGTGCTGAGCACCAGTCAGCATA | - |
| **A91H** | AGTATCAGGTTGGTGAGCGCACCAGTCAGCATA | - |
| **A91T** | AGTATCAGGTTGAGTTGCGCACCAGTCAGCATA | - |
| **T95A** | AGCACTCAGTTAACGTCGCATCAGGTTGTGCAGC | Hpa I |
| **T95I** | AGCACTCAGTTAAGGTGATATCAGGTTGTGCAGC | EcoR V |
| **T96A** | AGCACTCAGTGAGGGCAGTATCAGGTTGTGCAGC | *Dra* III |
| **T96I** | AGCACTCAGTGAGGATAGTATCAGGTTGTGCAGC | *Dra* III |
| **L97A** | TATGCAGCACTCAGTGGCGGTAGTATCAGGTTG | - |
| **T98A** | CTGTATGCAGCACTCGGCCAAGGTAGTATCAGGTTG | - |
| **T98V** | TATGCAGCACTCCACCAAGGTAGTATCAGGTTG | Sty I |
| **Q103A** | TCCAACATTCATCGCGATGCAGCACTCAG | *BstU* I |
| **N105A** | TCCAATTCCAACGGCCATCTGTATGCAGCA | *Eae* I |
| **G109D** | GTGTCATTCCAGCATTTATCGATTCCAACATTCATC | - |
| **T115A** | AAGATGCGTACAAGGCGCGTCATTCCAGCATTTTCC | - |
| **T115V** | AAGATGCGTACAAGGGACGTCATTCCAGCATTTTCC | Aat II |
| **H121A** | GTGGCTTGGCGCAGATTGAGCTCGCAAGATGCGTACAAGG | *Ban* II |

All primer sequences are from 5′ to 3′.
