# Supplementary material for: Structure and Evolutionary Origin of Ca2+-Dependent Herring Type II Antifreeze Protein
Source: PLoS One. 2007 Jun 20;2(6):e548. doi: 10.1371/journal.pone.0000548 (PMC1891086; doi:10.1371/journal.pone.0000548)
Supplement: Table S2 — Equilibrium dialysis assay of hAFP and its mutants (0.03 MB DOC) [file pone.0000548.s002.doc]

**Table S2.** Equilibrium dialysis assay of hAFP and its mutants

| **Protein name** | Protein concentration **(μM)** | K_d_ (μM) |
| --- | --- | --- |
| WT-6H | 45.2 | 34.2 ± 0.2 |
| **A91H** | 62.7 | 51.8 ± 0.8 |
| **A91T** | 45.7 | 34.1 ± 0.4 |
| **T96A** | 54.2 | 46.8 ± 0.3 |
| **T96I** | 71.1 | 33.0 ± 0.5 |
| **L97A** | 51.9 | 91.9 ± 1.0 |
| **T98A** | 57.2 | 58.7 ± 0.6 |
| **T98V** | 55.1 | 62.7 ± 0.4 |
| **T115A** | 68.3 | 77.4 ± 0.5 |
