# Supplementary material for: Structure and Evolutionary Origin of Ca2+-Dependent Herring Type II Antifreeze Protein
Source: PLoS One. 2007 Jun 20;2(6):e548. doi: 10.1371/journal.pone.0000548 (PMC1891086; doi:10.1371/journal.pone.0000548)
Supplement: Table S3 — Thermal hysteresis of solutions in the presence of hAFPs (0.07 MB DOC) [file pone.0000548.s003.doc]

**Table S3.** Thermal hysteresis of solutions in the presence of hAFPs

|  | **H_2_O** | **buffer** |  |  |  |  |  |  |  |
| --- | --- | --- | --- | --- | --- | --- | --- | --- | --- |
| Thermal hysteresis (°C) | 0* | 0.022 |  |  |  |  |  |  |  |
|  |  |  |  |  |  |  |  |  |  |
| **WT-6H** |  | **A90S** |  | **A90H** |  | **A91T** |  | **A91H** |  |
| protein concentration (mM) | Thermal hysteresis (°C)** | protein concentration (mM) | Thermal hysteresis (°C) | protein concentration (mM) | Thermal hysteresis (°C) | protein concentration (mM) | Thermal hysteresis (°C) | protein concentration (mM) | Thermal hysteresis (°C) |
| 0.098 | 0.17 | 0.104 | 0.152 | 0.096 | 0.151 | 0.098 | 0.13 | 0.088 | 0.074 |
| 0.197 | 0.217 | 0.208 | 0.198 | 0.193 | 0.186 | 0.196 | 0.167 | 0.177 | 0.108 |
| 0.295 | 0.248 | 0.312 | 0.22 | 0.29 | 0.22 | 0.294 | 0.186 | 0.265 | 0.136 |
| 0.393 | 0.307 | 0.416 | 0.276 | 0.387 | 0.238 | 0.392 | 0.22 | 0.353 | 0.158 |
| 0.491 | 0.341 | 0.52 | 0.282 | 0.483 | 0.276 | 0.49 | 0.242 | 0.441 | 0.18 |
| 0.59 | 0.359 | 0.624 | 0.334 | 0.58 | 0.3 | 0.588 | 0.285 | 0.53 | 0.191 |
|  |  |  |  |  |  |  |  |  |  |
| **G109D** |  | **H121A** |  | **Q103A** |  | **T95A** |  | **T95I** |  |
| protein concentration (mM) | Thermal hysteresis (°C) | protein concentration (mM) | Thermal hysteresis (°C) | protein concentration (mM) | Thermal hysteresis (°C) | protein concentration (mM) | Thermal hysteresis (°C) | protein concentration (mM) | Thermal hysteresis (°C) |
| 0.05 | 0.121 | 0.097 | 0.13 | 0.105 | 0.136 | 0.082 | 0.108 | 0.08 | 0.093 |
| 0.099 | 0.149 | 0.193 | 0.195 | 0.209 | 0.18 | 0.164 | 0.152 | 0.16 | 0.124 |
| 0.199 | 0.22 | 0.29 | 0.237 | 0.314 | 0.18 | 0.246 | 0.177 | 0.24 | 0.149 |
| 0.298 | 0.254 | 0.387 | 0.265 | 0.419 | 0.238 | 0.328 | 0.204 | 0.32 | 0.189 |
| 0.397 | 0.288 | 0.483 | 0.288 | 0.523 | 0.269 | 0.41 | 0.22 | 0.4 | 0.235 |
| 0.497 | 0.331 | 0.58 | 0.307 | 0.628 | 0.31 |  |  |  |  |
| 0.596 | 0.362 |  |  |  |  |  |  |  |  |
| * theoretical value | | | | | | | | | |
| ** Buffer contributions have been deducted from thermal hysteresis values in the presence of hAFPs. | | | | | | | | | |
|  |  |  |  |  |  |  |  |  |  |
