# Supplementary material for: Structure and Evolutionary Origin of Ca2+-Dependent Herring Type II Antifreeze Protein
Source: PLoS One. 2007 Jun 20;2(6):e548. doi: 10.1371/journal.pone.0000548 (PMC1891086; doi:10.1371/journal.pone.0000548)

**Figure S1.**  **Ca^2+^-induced intrinsic fluorescence changes of hAFP and its Ala mutants at positions 96, 97, 98, and 115.** Dashed lines represented the fluorescence spectra of apo-hAFPs, and solid lines represented the fluorescence spectra of hAFPs in the presence of Ca^2+^ ions. Ca^2+^ ions can induce intrinsic fluorescence changes of these Ala mutants, the same as that of the wild-type hAFP (WT-6H).


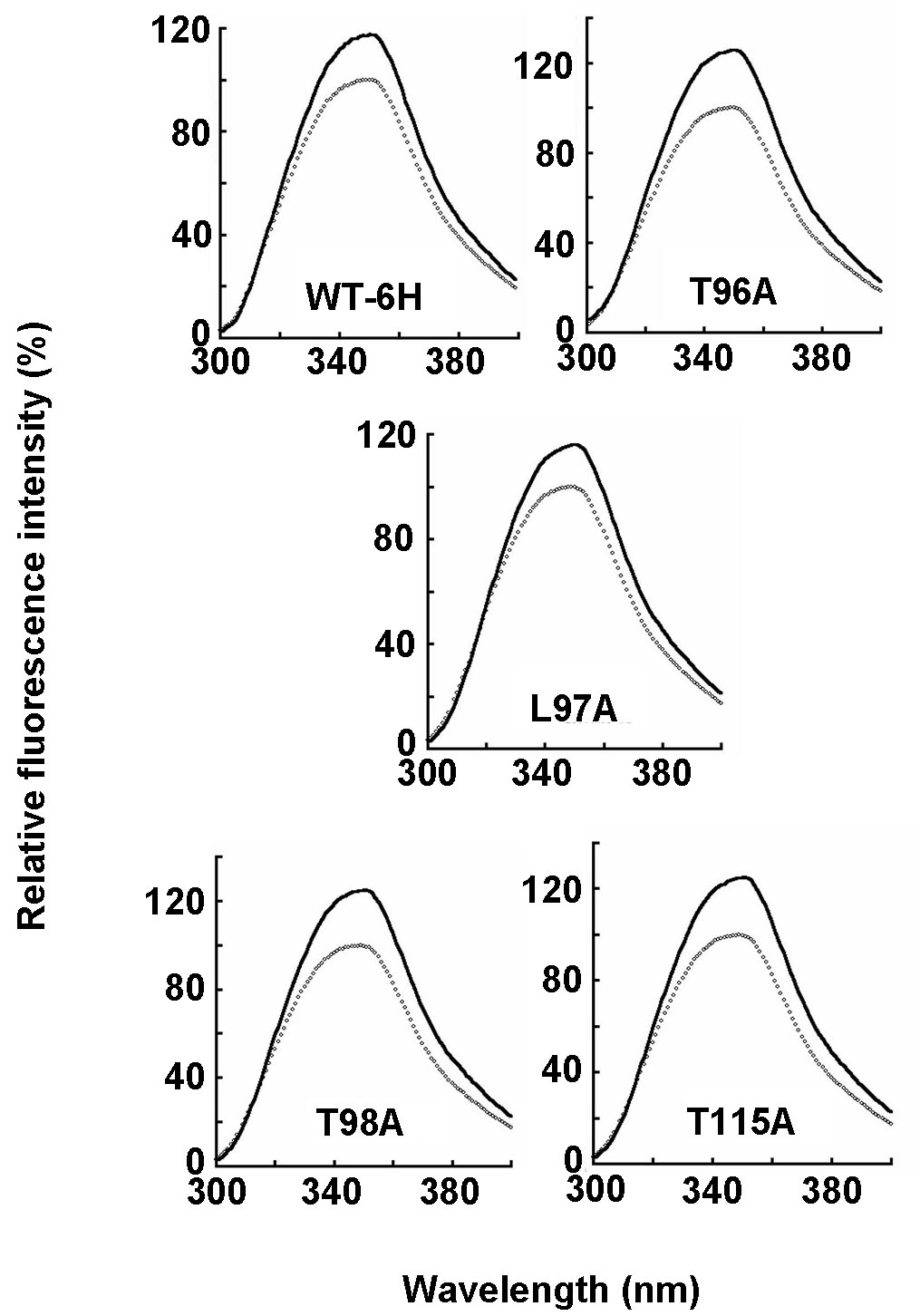

Supplement: Figure S1 — Ca2+-induced intrinsic fluorescence changes of hAFP and its Ala (0.18 MB DOC) [file pone.0000548.s004.doc]
