# Supplementary material for: Structure and Evolutionary Origin of Ca2+-Dependent Herring Type II Antifreeze Protein
Source: PLoS One. 2007 Jun 20;2(6):e548. doi: 10.1371/journal.pone.0000548 (PMC1891086; doi:10.1371/journal.pone.0000548)
Supplement: Figure S2 — Lattice planes of ice Ih, the hexagonal crystal form of ordinary ice. (0.07 MB DOC) [file pone.0000548.s005.doc]

**Figure S2. Lattice planes of ice I_h_, the hexagonal crystal form of ordinary ice.** The different ice lattice planes are indicated with arrows.
